# Xanthohumol Induces ROS through NADPH Oxidase, Causes Cell Cycle Arrest and Apoptosis

**DOI:** 10.1155/2021/9877170

**Published:** 2021-11-10

**Authors:** Chun-Ming Wang, Jun Chen, Jing Zhao, Shan-Shan Hu, Shu-Qiu Zhang, Xiang-Quan Mi, Xiang Shi, Xin-Hui Cao, Zhen Li

**Affiliations:** ^1^Ministry of Education Key Laboratory of Cell Activities and Stress Adaptations, School of Life Sciences, Lanzhou University, Lanzhou, 730000 Gansu, China; ^2^Second Hospital of Lanzhou University Dingxi Hospital (Dingxi City People's Hospital) Clinical Laboratory, Dingxi, 743000 Gansu, China

## Abstract

Reactive oxygen species (ROS) are either toxic in excess or essential for redox signalling at the physiological level, which is closely related to the site of generation. Xanthohumol (XN) is an important natural product of hops (*Humulus lupulus* L.) and was reported to induce ROS in mitochondria. While in the present study, our data indicate that NADPH oxidase (NOX) is another site. In human acute myeloid leukemia HL-60 cells, we first identified that cell proliferation was inhibited by XN without affecting viability, and this could be alleviated by the antioxidant N-acetyl-L-cysteine (NAC); cell cycles were blocked at G1 phase, apoptosis was induced in a dose-dependent manner, and malondialdehyde (MDA) content was upregulated. XN-induced ROS generation was detected by flow cytometry, which can be inhibited by diphenyleneiodonium chloride (DPI, a NOX inhibitor), while not by N^G^-methyl-L-arginine acetate (L-NMMA, a nitric oxide synthase inhibitor). The involvement of NOX in XN-induced ROS generation was further evaluated: immunofluorescence assay indicated subunits assembled in the membrane, and gp91^phox^ knockdown with siRNA decreased XN-induced ROS. Human red blood cells (with NOX, without mitochondria) were further selected as a cell model, and the XN-induced ROS and DPI inhibiting effects were found again. In conclusion, our results indicate that XN exhibits antiproliferation effects through ROS-related mechanisms, and NOX is a source of XN-induced ROS. As NOX-sourced ROS are critical for phagocytosis, our findings may contribute to the anti-infection and anti-inflammatory effect of XN.

## 1. Introduction

ROS play a dual role in the initiation, development, suppression, and treatment of cancer [1, 2], and many anticancer drugs are efficient to eliminate cancer cells and drug resistance by increasing ROS production [2]. Natural products have played a key role in drug discovery [3, 4], especially for cancer, including acute myeloid leukemia [5]. So, it is important to evaluate the effect of natural products on ROS generation and the related mechanisms.

Xanthohumol (XN) is the major prenylflavonoid of hops (*Humulus lupulus* L.), which may constitute 0.1-1% of dry weight [6]. Many potential biological activities of XN have been reported, such as anti-infection against microorganisms including bacteria, viruses, fungi, and malarial protozoa [7], and anti-inflammatory [8], as well as the most widely studied anticancer properties [9–11]. Most of these activities correlate with the ROS-related mechanisms [9–15]. And, as a flavonoid, its antioxidant activities were widely investigated in the early time [16], while the prooxidant effects are mostly reported in the recent years [17–19]. About the source of ROS induced by XN, it was reported, early in the year 2010, that XN induced superoxide anion radical formation via mitochondria [20], and another group further pointed out that XN induced ROS through inhibition of mitochondrial electron transfer chain complex I [18]. While in the present study, we found that NADPH oxidase (NOX) is also a source of XN-induced ROS.

NOX is first found in phagocytes and further identified in a wide variety of nonphagocytic cells. It belongs to a family of seven members (NOX1-5, DUOX1, and 2). As a multicomponent enzyme complex, NOX consists of membrane-bound cytochrome b-558 (a heterodimer of gp91^phox^ and p22^phox^) and the cytosolic regulatory subunits p47^phox^, p67^phox^, p40^phox^, and the small GTP-binding protein Rac1. The presentation of NOX in human acute myeloid leukemia HL-60 cells and human red blood cells (RBCs) is well established [21–23]; thus, HL-60 and RBCs are used as cell models in the present study.

NOX produces superoxide anion through transferring electrons from NADPH to molecular oxygen, which is rapidly converted into hydrogen peroxide. These large amounts of ROS in phagosomes function phagocytes to kill ingested microbes [24]. In addition, NOX also plays an important role in immunomodulation [25]. That is to say, NOX-derived ROS may shape the regulation of the adaptive immune response and play a role in the resolution of the inflammatory response [24]. On the other hand, acceleration of accumulative ROS may disrupt redox homeostasis and cause severe damage in cancer cells, and this is the mechanism of anticancer therapy of many prooxidative anticancer drugs such as 2-methoxyestradiol, buthionine sulfoximine, cisplatin, doxorubicin, imexon, and motexafin gadolinium [26]. Thus, the prooxidant activity of XN may support its anti-infection, anti-inflammatory, and anticancer properties.

We have reported ROS generation [17] and paraptosis induction effect induced by XN [27] in HL-60 cells. In the present study, using the same HL-60 cells and human RBCs, we show that NOX is also the source of XN-induced ROS.

## 2. Materials and Methods

### 2.1. Chemicals and Reagents

Xanthohumol was provided by Yumen Tuopu Scientific and Technological Development Co., Ltd., purity > 98% (Yumen, Gansu, China). *N*-Acetylcysteine (NAC), propidium iodide (PI), 2′,7′-dichlorofluorescin diacetate (DCFH-DA), dihydroethidium (DHE), diphenyleneiodonium chloride (DPI), and N^G^-methyl-L-arginine acetate salt (L-NMMA) were purchased from Sigma-Aldrich (St. Louis, MO, USA). The cell culture medium RPMI-1640 was purchased from Invitrogen (Carlsbad, CA, USA). Neonatal bovine serum was purchased from Lanzhou Minhai Bio-engineering Co., Ltd. (Lanzhou, China). RIPA Lysis Buffer and Enhanced BCA Protein Assay Kit were purchased from Beyotime (Shanghai, China).

### 2.2. Cell Culture

Human acute myeloid leukemia cell line HL-60 was obtained from the cell bank of Chinese Academy of Science (Shanghai, China) and stored in liquid nitrogen in our own lab. Cells were maintained in RPMI-1640 medium supplemented with 10% inactivated super neonatal bovine serum, 100 U/ml penicillin, 100 *μ*g/ml streptomycin, and 2.0 mg/ml NaHCO_3_ at 37°C with 5% CO_2_ in a humidified atmosphere. Cells under exponential growth phase cultures were harvested, counted, and seeded in 12.5 cm^2^ cell culture flasks (1 × 10^6^ cells/flask) in the experiments described below.

### 2.3. Trypan Blue Exclusion Assay

After seeded in flasks, cells were treated with different concentrations of XN with or without 1 mM NAC for 24 h. Then, cells were collected, and the trypan blue exclusion assay was performed to determine the effects of XN on cell proliferation and cell viability according to our previously described method [17]. Cell viability is defined as the number of healthy cells in a sample. And generally, methods used to determine viability are also common for the detection of cell proliferation [28]. The trypan blue exclusion assay was developed as both proliferation and cytotoxicity assays [29]. Cell viability (cytotoxicity) is the percentage of the cells without stained with trypan blue in a sample. Cell proliferation can be calculated with number of cells after treatment compared with the seeding number.

### 2.4. Cell Cycle Analysis

After treated with different concentrations of XN for 24 h, the cell cycle was assessed with PI staining and flow cytometry assay as described previously [30].

### 2.5. Annexin V–FITC/PI Staining Assay

After treated with XN for 24 h, cell apoptosis was evaluated with an Annexin V-FITC Apoptosis Detection Kit purchased from eBioscience Inc. (San Diego, CA, USA) using a flow cytometer (BD LSRFortessa) as described previously [17].

### 2.6. Microscale Malondialdehyde (MDA) Assay

MDA content was detected with the microscale malondialdehyde assay kit (thiobarbituric acid (TBA) method) purchased from Nanjing Jiancheng Bioengineering Institute (Nanjing, China). In brief, after treated with XN for 24 h, cells were collected, washed, and resuspended in PBS to form a 1 × 10^7^ cells/ml suspension. Then, the cells were ultrasonic crushed and centrifuged at 4°C, 3500 rpm for 10 min to collect the supernatant, which was further processed following the instruments of MDA assay kit. Finally, the absorbance of the red TBA-MDA complex was measured at 532 nm, and the MDA content (nmol/mg protein) was calculated. Protein concentrations were measured with the Enhanced BCA Protein Assay Kit.

### 2.7. Intracellular ROS Generation Detected with DCFH-DA or DHE

Intracellular ROS levels were assessed by measuring the oxidative conversion of cell permeable DCFH-DA to fluorescent dichlorofluorescein (DCF) as described previously [17]. In brief, before treated with XN, cells were incubated with 5 *μ*M DCFH-DA for 45 min (loaded with the florescent probe), then washed with PBS (500 × *g*, 5 min at room temperature), resuspended in RPMI-1640, and then treated with XN for 0.5, 1, or 2 h. Cells were then washed, resuspended in 500 *μ*l PBS, and analyzed immediately by flow cytometry. DPI (NADPH oxidase inhibitor) was incubated with cells at the same time with DCFH-DA, when evaluating the effect of DPI on XN-induced ROS generation. When detecting the ROS in RBCs, 20 *μ*M DHE was used.

### 2.8. Immunofluorescence (IF) Assay

After treatment, HL-60 cells were collected by centrifugation, washed with PBS, fixed with paraformaldehyde, and hybridized with primary antibodies (gp91-^phox^ (H-60), sc-20782; p47-^phox^ (C-20), sc-7660; Santa Cruz, CA, USA) overnight at 4°C. The cells were then washed with PBS and probed with fluorophore-conjugated secondary antibodies (bovine anti-rabbit IgG-TR, sc-2787; donkey anti-goat IgG-FITC, sc-2024), respectively. The fluorescent images were obtained with a Leica confocal microscope (LeicaTCS SP8).

### 2.9. siRNA-Mediated gp91^phox^ Gene Silencing

siRNA against gp91^phox^ (sc-35503), related RT-PCR Primer (sc-35503-PR), Control siRNA (sc-37007), and siRNA Reagent System (sc-45064) were purchased from Santa Cruz Biotechnology, Inc. (Santa Cruz, CA, USA); transfections were performed by using the methods and reagents described by the manufacturer.

### 2.10. Blood Samples and Red Blood Cells (RBCs) Preparation and Incubation

Blood was obtained from healthy adult volunteers and approved by the Ethics Committee of School of Life Sciences, Lanzhou University. After collection, 2 ml of K_2_EDTA-anticoagulated venous blood samples was centrifuged at 500 × g for 5 min at room temperature; the supernatant was discarded, and the cells were washed twice in PBS to remove residual plasma and white blood cells in the top cell layer. RBCs were resuspended at 0.2% haematocrit in RPMI-1640 (R6504, Sigma) and incubated with DHE (D7008, Sigma) at 20 *μ*M for 30 min at 37°C. Then, cells were treated with XN, and DPI was preincubated for 1 h before XN was treated when evaluating the role of NOX. After treatment, RBCs were collected, resuspended in PBS, and analyzed immediately by flow cytometry.

### 2.11. Statistical Analysis

Results were expressed as the mean ± SD from at least three independent experiments. Statistical analysis was conducted using Microsoft Excel 2010 (Microsoft Corp). Statistical differences were determined using the two-tailed Student's *t*-test for comparisons between two groups. Statistically significant differences are indicated by ∗, ∗∗, and ∗∗∗ where *p* values < 0.05, 0.01, and 0.001 vs. the control group, respectively, and by #, ##, and ### where *p* < 0.05, 0.01, and 0.001 vs. indicated groups, respectively.

## 3. Results

### 3.1. XN Inhibits Cell Proliferation, Induces G1 Phase Cell Cycle Arrest and Apoptosis, Which Is Related to Oxidative Stress

**Figure 1:**
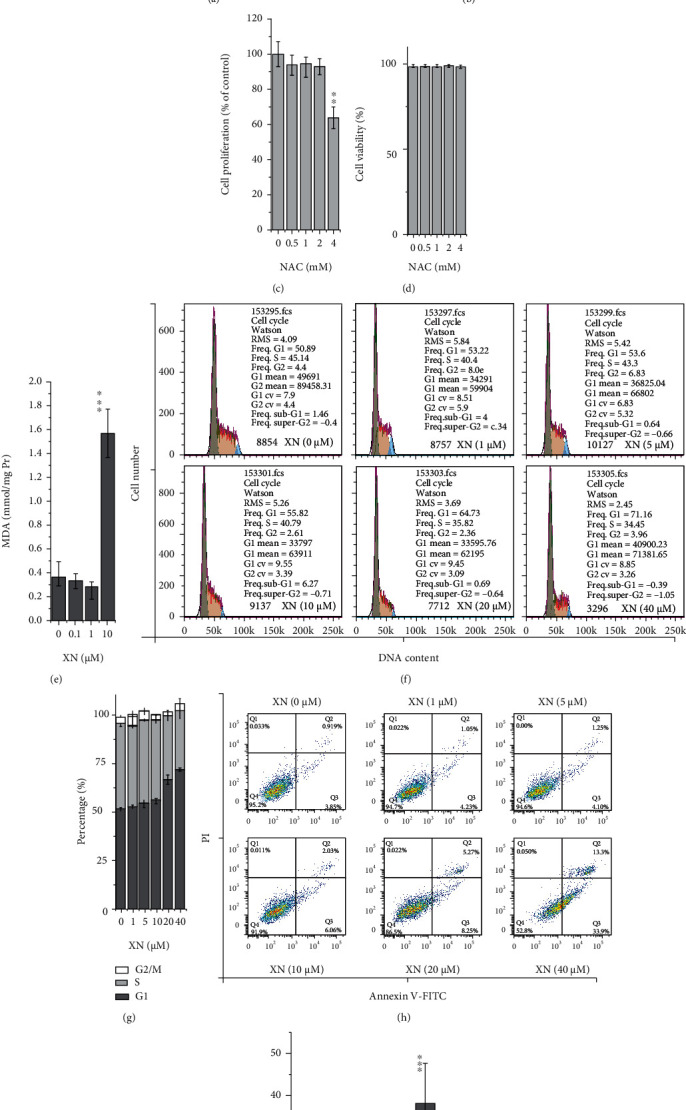
The effect of XN on cell proliferation, cell viability, cell cycle, and apoptosis: (a–d) cell proliferation and viability detected with trypan blue exclusion assay. HL-60 cells were treated with XN (with or without 30 min preincubated with 1 mM NAC) or NAC for 24 h (*n* = 3; ^∗^*p* < 0.05, ^∗∗^*p* < 0.01, ^∗∗∗^*p* < 0.001 vs. control; ##*p* < 0.01, “XN” vs. “XN + NAC”); (e) MDA content of cells after treated with XN for 24 h (*n* = 3; ^∗∗∗^*p* < 0.001 vs. control); (f, g) the effect of XN on cell cycle distribution after treated for 24 h (f) and the related statistical results (g); (h, i) apoptosis evaluated with Annexin V–FITC/PI staining assay after treated with XN for 24 h.

XN inhibited cell proliferation in HL-60 cells in a dose-dependent manner, which could be alleviated by preincubation with 1 mM NAC (Figure 1(a)). There was no cell proliferation inhibition or cytotoxicity induction activity exhibited when treated with 1 mM NAC alone for 24 h (Figures 1(c) and 1(d)). These results indicated that XN inhibited cell proliferation through an oxidative stress-related mechanism, and this was further confirmed by the elevated MDA (lipid peroxidation biomarker of oxidative stress [31]) content under 10 *μ*M XN treatment (Figure 1(e)).

Cell viability determined by the trypan blue exclusion test represents the percentage of cells that have clear cytoplasm (viable cells). XN treatment did not affect cell viability, neither in the XN treated groups nor in the coincubation groups with NAC (Figure 1(b)). This indicates that the inhibited effect on cell proliferation is not through cytotoxicity. Therefore, cell cycle and apoptosis were further evaluated. The results indicated that G1 phase cell cycle arrest (Figures 1(f) and 1(g)) and apoptosis (Figures 1(h) and 1(i)) were both induced by XN in a dose-dependent manner. This may indicate that XN inhibits cell proliferation through inducing cell cycle arrest and apoptosis, rather than directly affecting cell viability.

### 3.2. XN Induces ROS Generation Which Can Be Inhibited by DPI but Not L-NMMA

**Figure 2:**
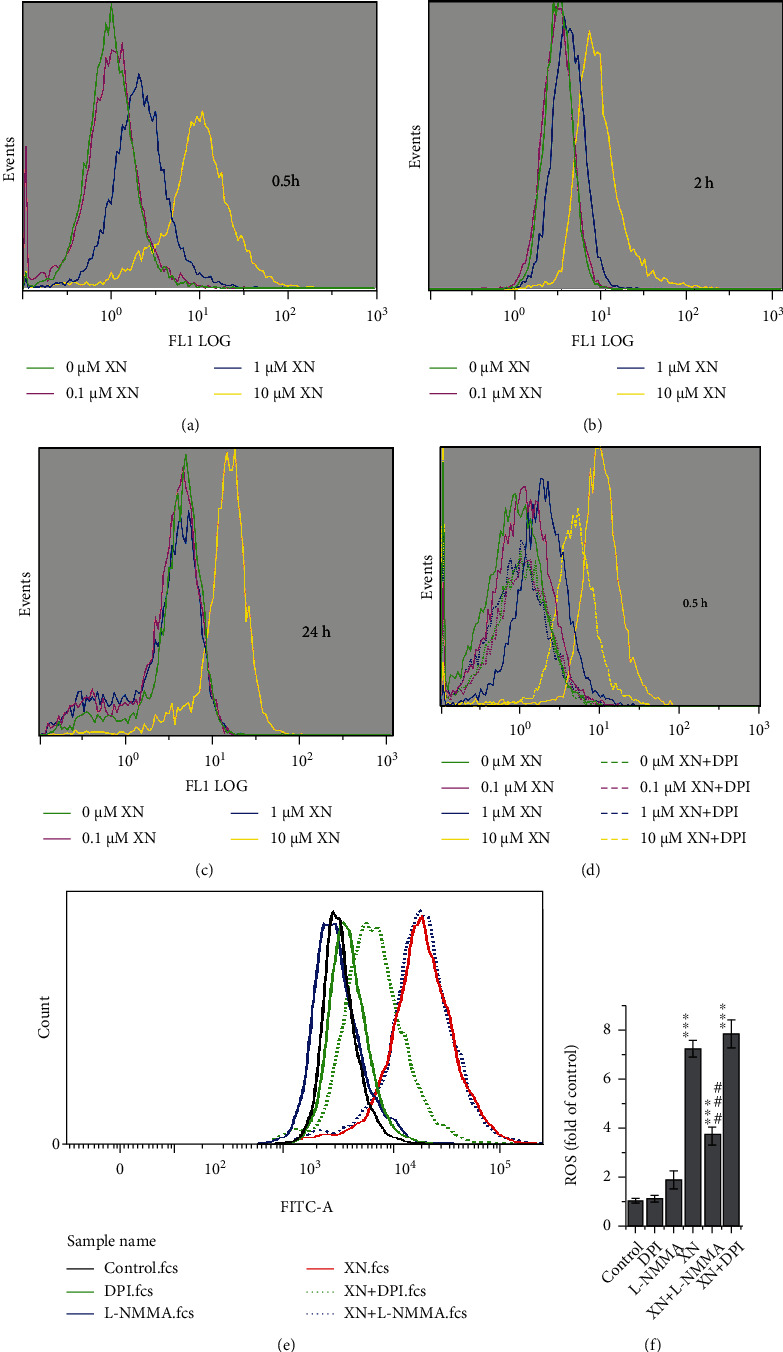
XN induces ROS generation and the role of NOX evaluated with chemical inhibitors. (a–c) Flow cytometry histograms showed ROS generated in HL-60 cells after XN treated for 0.5, 2, and 24 h. (d) The effect of DPI (10 *μ*M) on ROS after treated with XN for 0.5 h. (e, f) One representative experiment of the effect of DPI (10 *μ*M) and L-NMMA (100 *μ*M) on ROS after treated with 10 *μ*M XN for 1 h (e) and the related statistical results (f) (*n* = 3; ^∗∗∗^*p* < 0.001 vs. control; ###*p* < 0.001, “XN + DPI” vs. “XN”).

In view that the antioxidant NAC alleviates cell proliferation inhibition effect of XN (Figure 1(a)), it is reasonable to connect the related mechanisms with ROS generation. Thus, the effects of XN on ROS generation were evaluated. The representative flow cytometry histograms showed that XN induced ROS generation in a dose-dependent manner (Figures 2(a)–2(c)). Both 1 and 10 *μ*M XN induced an apparent increase of ROS, especially after treated for 0.5 and 2 h (Figures 2(a) and 2(b)). While when estimating the time-dependent trend, it is clear that ROS induced by lower concentrations of XN (such as 1 *μ*M) decreased after treated for 2 h compared to 0.5 h and further decreased to the control level after treated for 24 h (Figures 2(a)–2(c)), and only the higher concentration of XN (10 *μ*M) kept on exhibiting ROS induction effect after 24 h treatment (Figure 2(c)). This is consistent with its cell proliferation inhibiting function shown in Figure 1(a).

As NOX locates in the cell membrane, it ought to be the first component which encounters XN, so we further evaluated the role of NOX in XN-induced ROS. As shown in Figure 2(d), 10 *μ*M DPI, a NOX inhibitor, significantly inhibited ROS generation under all three XN concentrations (0.1, 1, and 10 *μ*M). The ROS levels induced by 0.1 and 1 *μ*M XN were even decreased to the control group level (Figure 2(d)).

Because DCFH-DA reacts with not only H_2_O_2_ but also nitric oxide [32] and DPI inhibits both NOX and nitric oxide synthase (NOS) activities [33, 34], it is hard to say that the inhibition effect of DPI on XN induced DCF fluorescence was contributed to its inhibiting NOX or NOS. Thus, we further used an NOS inhibitor L-NMMA, to discriminate the role of NOX and NOS in XN induced ROS generation. The results showed that it was DPI but not L-NMMA inhibited XN-induced ROS (Figures 2(e) and 2(f)), which indicated that XN induced ROS through NOX but not NOS. This result has also found in the HeLa cells (Fig. S1).

We checked the effect of XN treatment on gp91^phox^ and p47^phox^ (NOX subunit) expression with Western blot assay. Only gp91^phox^ was found upregulated under treatment with XN, while p47^phox^ was not (Fig. S2).

### 3.3. XN Induces NOX Activation

**Figure 3:**
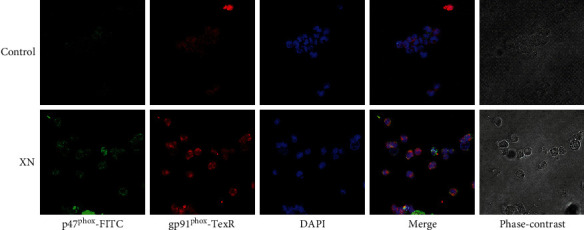
Representative images showing XN-induced colocalization between NOX subunits gp91^phox^ and p47^phox^. After treated with 10 *μ*M XN for 1 h, cells were collected, washed, fixed, and incubated with gp91^phox^ and p47^phox^ primary antibodies overnight at 4°C and then probed with TR and FITC conjugated secondary antibodies, respectively. The fluorescent images were obtained with a confocal microscope (200x).

When activating, the cytosolic subunits of NOX (p47^phox^, p67^phox^, p40^phox^, and Rac1) will translocate to the plasma membrane and form an active complex with the membrane subunits (gp91^phox^ and p22^phox^). If XN induces ROS through NOX, this translocation should happen. Thus, we studied the colocalization of gp91^phox^ with p47^phox^ using immunofluorescence assay. The results showed that colocalization happened indeed after treated with XN compared to the control cells (Figure 3).

### 3.4. NOX Subunit gp91^phox^ Gene Knockdown Decreases XN-Induced ROS

**Figure 4:**
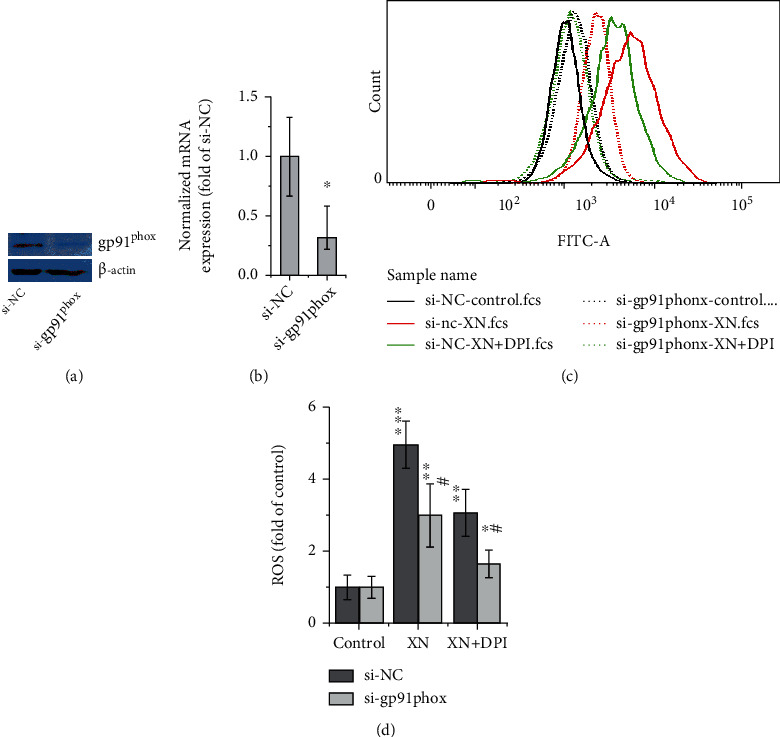
The effect of gp91^phox^ gene knockdown on XN-induced ROS. HL-60 cells were transfected with gp91^phox^ siRNA (si-gp91^phox^) or its nontargeting control (si-NC) using methods and reagents described by the manufacturer (Santa Cruz, CA, USA) and incubated for an additional 24 hours, then treated with 10 *μ*M XN for 1 h. Western blot (a) and QRT-PCR data (b) showed the gp91^phox^ protein and mRNA levels after transfected with siRNA, respectively (a, b). A representative flow cytometry histogram (c) and the related statistical results (d) were presented to show the effect of XN and DPI on HL-60 cells' ROS generation (*n* = 3; ^∗^*p* < 0.05, ^∗∗^*p* < 0.01, ^∗∗∗^*p* < 0.001 vs. control; #*p* < 0.05, “si-NC vs. si-gp91^phox^”).

To confirm the role of NOX on XN-induced ROS generation found above with chemical inhibitors (Figures 2(d)–2(f)) and immunofluorescence confocal images (Figure 3), we further evaluated the effect of NOX subunit gp91^phox^ gene knockdown with RNAi method. The results indicated that, compared with the related control cells, cells with gp91^phox^ gene knockdown exhibited a significant decrease in XN-induced ROS (Figure 4). What surprisingly, the NOX inhibitor DPI still exhibited the inhibition effect in the gene-manipulated cells. We wondered whether it is related to the reports that XN induces ROS through mitochondria [18, 20], because it is reported that DPI is not a specific inhibitor of NOX, which may also inhibit ROS generated at mitochondria and other sites [34]. So, we further examined the effect of DPI on human RBCs, which lack mitochondria while still have NOX.

### 3.5. XN Induces ROS Generation in RBCs and Which Can Be Inhibited by DPI

**Figure 5:**
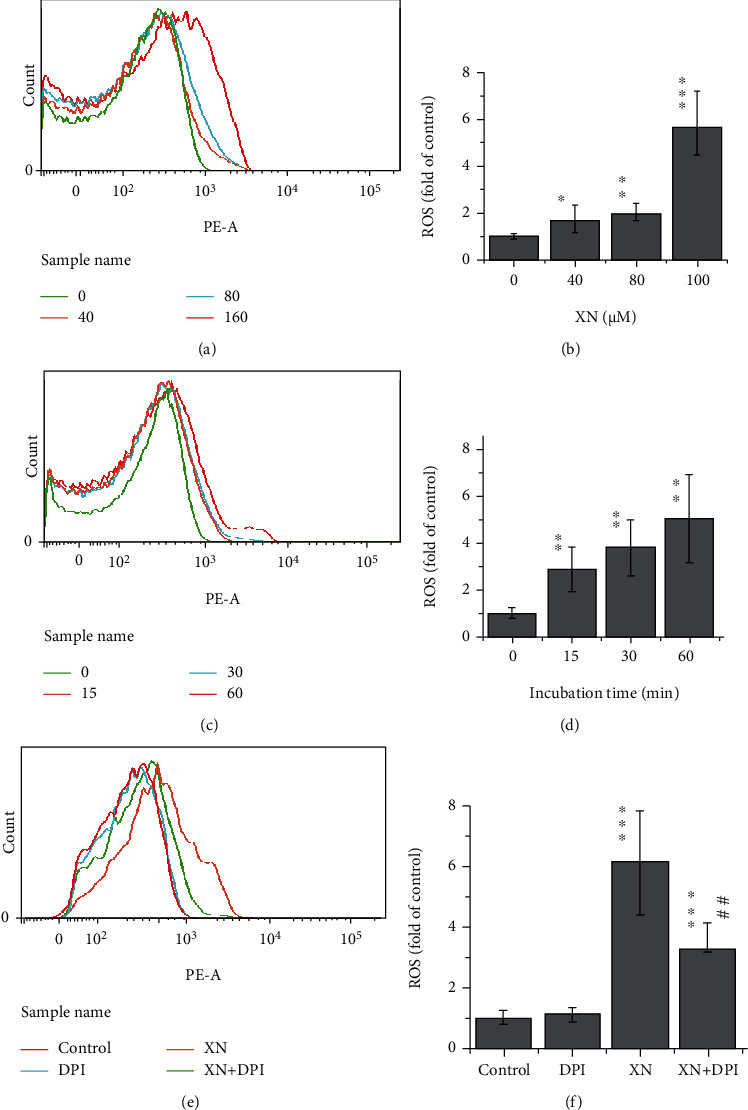
The effect of XN and DPI on ROS generation in RBCs. Human RBCs lack mitochondria while having NOX. (a, c, e) Representative flow cytometry histograms show dose- and time-dependent effect of XN on ROS in RBCs and the effect of DPI, respectively. (b, d, f) The related statistical results, respectively. RBCs were treated with XN for 1 h in (a, b, e, f), and the XN concentration used in (c–f) was 160 *μ*M (*n* = 3, ^∗^*p* < 0.05, ^∗∗^*p* < 0.01, and ^∗∗∗^*p* < 0.001 vs. control group; ##*p* < 0.01 “XN vs. XN+DPI”).

Human RBCs lack mitochondria but still have NOX [23]. Therefore, it is a good cell model to use RBCs for studying the effect of DPI on XN-induced ROS. The results indicated that XN can induce ROS in RBCs in both a dose-dependent (Figures 5(a) and 5(b)) and a time-dependent (Figures 5(c) and 5(d)) manner, which can also be inhibited by the NOX inhibitor DPI (Figures 5(e) and 5(f)). As shown in Figure 5(f), DPI cannot completely inhibit XN-induced ROS in RBCs to the control level. This cannot attribute to the inhibition of mitochondria by DPI, for RBCs lack mitochondria. It is hard to explain based on the present data, but it is a common result that DPI cannot inhibit ROS levels to the control group level in many other studies [35, 36].

## 4. Discussion

In the present study, we found the oxidative stress-related antiproliferation activity of XN in HL-60 cells. The antiproliferation effect is not directly attributed to cytotoxicity but through inducing cell cycle arrest and apoptosis. More impressively, this property is correlated with the ROS induction activity of XN. Evidences include that NAC alleviated the antiproliferation effect of XN, and XN treatment increased MDA content and ROS generation. By using chemical inhibitors (DPI and L-NMMA), immunofluorescence observation, siRNA knocking down gp91^phox^ gene, and the RBCs cell model, the role of NOX in XN-induced ROS generation is established.

The anticancer activity of XN has been widely studied, and many reports are related with ROS [9–11]. One of the antileukemia studies mentioned the elevation of ROS caused by XN in K562 cells [37]. But the NAC concentration used in that study was too high (10 mM) compared to the other studies treating K562 cells with NAC (0.1–2.5 mM) [38–41]. Our data showed that there was no effect on cell proliferation under 2 mM NAC treatment, while the results were only 60% of control group when treated with 4 mM NAC (Figure 2(c)). Cell cycle arrest and apoptosis are two mechanisms frequently studied about the anticancer effect of XN [9–11]. XN induced cell cycle arrest at G1, S, and G2/M phases in different cancer cells [9–11]. A G2/M phase arrest and apoptosis were reported in a human colon carcinoma recently [42]. We report a G1 phase arrest in HL-60 cells in the present study (Figures 1(f) and 1(g)). The different cell cycle arrest induced by XN may be related to the different cancer cells used in the studies.

Although it has been reported that XN induced ROS through mitochondria [20] and even inhibited the activity of mitochondrial complex I [18], our data proved that XN induced ROS generation also through NOX. In view of the fact that XN still induces ROS even in gp91^phox^ knockdown HL-60 cells (Figure 4) and DPI cannot inhibit the XN-induced ROS to the basal level in human RBCs (Figures 5(e) and 5(f)), it is reasonable to conclude that XN may be a multisite ROS activator.

The sources of ROS are closely correlated with their functions. Among the two main sources of ROS in cells, mitochondria, and NOX, mitochondria-sourced ROS are generally thought to relate to cell damage and aging, while ROS from NOX are either responsible for the antibacterial behaviour in phagocytes during the respiratory burst or involve in signalling transduction in other cells. In recent years, crosstalk between mitochondria and NOX via ROS signalling is getting increasing attention [43, 44], which was termed “ROS-induced ROS release,” may amplify ROS generation in different subcellular compartments and indicates that both ROS sources are important in signalling function.

The content of ROS is also an important factor in determining whether they exhibit the oxidative stress damage effect or the signalling function. Higher ROS levels usually cause damage, while lower levels may tend to signal function. According to our data, ROS induced by XN are in a dose-dependent manner, and ROS induced by the lower doses (0.1 and 1 *μ*M) decreased to the control levels after treated for 24 h, while the higher concentration XN (10 *μ*M) kept on inducing ROS even after 24 h incubation (Figures 2(a)–2(c)). Consistent with this, 10 *μ*M XN induced MDA increased while 0.1 and 1 *μ*M XN did not (Figure 1(e)). This may indicate that XN exhibits signalling function in lower concentrations, while causing damage under higher concentrations.

XN is the major prenylflavonoid in hops, but it is generally a minor prenylflavonoid in beer due to thermal isomerization of chalcones into flavanones during the brewing process [6]. Thus, it is no need to worry about the detrimental health effects due to dietary exposure through beer consumption. On the other hand, the prooxidant property of XN may contribute to the anti-infection, anti-inflammatory, and anticancer drug development [9].

## 5. Conclusions

Collectively, we identified for the first time that XN induces ROS generation through NOX, which was mainly supported by the results of chemical inhibitors, siRNA of gp91^phox^, NOX subunit assembling, and RBC model. This may help to explain the anti-infection, anti-inflammatory, and anticancer activities of XN.

## Data Availability

The data are available on request from the corresponding author, Chun-Ming Wang, through email: cmwang@lzu.edu.cn.

## Supplementary Materials

Supplementary Materials Figure S1: the effect of DPI (10 *μ*M) and L-NMMA (100 *μ*M) on ROS generation in HeLa cells after 10 *μ*M XN treated for 1 h (*n* = 3; ^∗∗∗^*p* < 0.001 vs. control; ###*p* < 0.001, “XN + DPI” vs. “XN”). Figure S2: the effect of XN on gp91^phox^ and p47^phox^ expression after 24 h treatment detected with Western blot and analyzed with the ImageJ software. HL-60 cells were treated with different concentrations of XN for 24 h (*n* = 4; ^∗^*p* < 0.05 vs. control).

## References

[1] Huang R., Chen H., Liang J. (2021). Dual role of reactive oxygen species and their application in cancer therapy. *Journal of Cancer*.

[2] Kirtonia A., Sethi G., Garg M. (2020). The multifaceted role of reactive oxygen species in tumorigenesis. *Cellular and Molecular Life Sciences*.

[3] the International Natural Product Sciences Taskforce, Atanasov A. G., Zotchev S. B., Dirsch V. M., Supuran C. T. (2021). Natural products in drug discovery: advances and opportunities. *Nature Reviews. Drug Discovery*.

[4] Newman D. J., Cragg G. M. (2020). Natural products as sources of new drugs over the nearly four decades from 01/1981 to 09/2019. *Journal of Natural Products*.

[5] Kirtonia A., Pandya G., Sethi G., Pandey A. K., Das B. C., Garg M. (2020). A comprehensive review of genetic alterations and molecular targeted therapies for the implementation of personalized medicine in acute myeloid leukemia. *Journal of Molecular Medicine (Berlin, Germany)*.

[6] Stevens J. F., Page J. E. (2004). Xanthohumol and related prenylflavonoids from hops and beer: to your good health!. *Phytochemistry*.

[7] Gerhauser C. (2005). Broad spectrum antiinfective potential of xanthohumol from hop (Humulus lupulus L.) in comparison with activities of other hop constituents and xanthohumol metabolites. *Molecular Nutrition & Food Research*.

[8] Khayyal M. T., el-Hazek R. M., el-Sabbagh W. A., Frank J., Behnam D., Abdel-Tawab M. (2020). Micellar solubilization enhances the anti-inflammatory effect of xanthohumol. *Phytomedicine*.

[9] Jiang C. H., Sun T. L., Xiang D. X., Wei S. S., Li W. Q. (2018). Anticancer activity and mechanism of xanthohumol: a prenylated flavonoid from hops (Humulus lupulus L.). *Frontiers in Pharmacology*.

[10] Girisa S., Saikia Q., Bordoloi D. (2021). Xanthohumol from hop: hope for cancer prevention and treatment. *IUBMB Life*.

[11] Harish V., Haque E., Śmiech M. (2021). Xanthohumol for human malignancies: chemistry, pharmacokinetics and molecular targets. *International Journal of Molecular Sciences*.

[12] Magalhães P. J., Carvalho D. O., Cruz J. M., Guido L. F., Barros A. A. (2009). Fundamentals and health benefits of xanthohumol, a natural product derived from hops and beer. *Natural Product Communications*.

[13] Liu M., Hansen P., Wang G. (2015). Pharmacological profile of xanthohumol, a prenylated flavonoid from hops (Humulus lupulus). *Molecules*.

[14] Weiskirchen R., Mahli A., Weiskirchen S., Hellerbrand C. (2015). The hop constituent xanthohumol exhibits hepatoprotective effects and inhibits the activation of hepatic stellate cells at different levels. *Frontiers in Physiology*.

[15] Kirkwood J. S., Legette L. C. L., Miranda C. L., Jiang Y., Stevens J. F. (2013). A metabolomics-driven elucidation of the anti-obesity mechanisms of xanthohumol. *The Journal of Biological Chemistry*.

[16] Dietz B. M., Kang Y. H., Liu G. (2005). Xanthohumol isolated from Humulus lupulus inhibits menadione-induced DNA damage through induction of quinone reductase. *Chemical Research in Toxicology*.

[17] Wang C. M., Huo X., Chen J. (2020). An acute lytic cell death induced by xanthohumol obstructed ROS detecting in HL-60 cells. *Toxicology In Vitro*.

[18] Zhang B., Chu W., Wei P., Liu Y., Wei T. (2015). Xanthohumol induces generation of reactive oxygen species and triggers apoptosis through inhibition of mitochondrial electron transfer chain complex I. *Free Radical Biology & Medicine*.

[19] Festa M., Capasso A., D’Acunto C. W. (2011). Xanthohumol induces apoptosis in human malignant glioblastoma cells by increasing reactive oxygen species and activating MAPK pathways. *Journal of Natural Products*.

[20] Strathmann J., Klimo K., Sauer S. W., Okun J. G., Prehn J. H. M., Gerhäuser C. (2010). Xanthohumol-induced transient superoxide anion radical formation triggers cancer cells into apoptosis via a mitochondria-mediated mechanism. *The FASEB Journal*.

[21] Miyamoto D., Someya A., Nunoi H., Nagaoka I., Yamashita T. (1994). Analysis of the NADPH oxidase components during differentiation of HL-60 cells to eosinophilic lineage. *Biochimica et Biophysica Acta*.

[22] Bréchard S., Plançon S., Melchior C., Tschirhart E. J. (2009). STIM1 but not STIM2 is an essential regulator of Ca^2+^ influx-mediated NADPH oxidase activity in neutrophil-like HL-60 cells. *Biochemical Pharmacology*.

[23] George A., Pushkaran S., Konstantinidis D. G. (2013). Erythrocyte NADPH oxidase activity modulated by Rac GTPases, PKC, and plasma cytokines contributes to oxidative stress in sickle cell disease. *Blood*.

[24] Moghadam Z. M., Henneke P., Kolter J. (2021). From flies to men: ROS and the NADPH oxidase in phagocytes. *Frontiers in Cell and Development Biology*.

[25] Trevelin S. C., Shah A. M., Lombardi G. (2020). Beyond bacterial killing: NADPH oxidase 2 is an immunomodulator. *Immunology Letters*.

[26] Kim S. J., Kim H. S., Seo Y. R. (2019). Understanding of ROS-inducing strategy in anticancer therapy. *Oxidative Medicine and Cellular Longevity*.

[27] Mi X., Wang C., Sun C. (2017). Xanthohumol induces paraptosis of leukemia cells through p38 mitogen activated protein kinase signaling pathway. *Oncotarget*.

[28] Adan A., Kiraz Y., Baran Y. (2016). Cell proliferation and cytotoxicity assays. *Current Pharmaceutical Biotechnology*.

[29] Mosmann T. (1983). Rapid colorimetric assay for cellular growth and survival: application to proliferation and cytotoxicity assays. *Journal of Immunological Methods*.

[30] Pu L. P., Chen H. P., Cao M. A. (2013). The antiangiogenic activity of Kushecarpin D, a novel flavonoid isolated from Sophora flavescens Ait. *Life Sciences*.

[31] Tsikas D. (2017). Assessment of lipid peroxidation by measuring malondialdehyde (MDA) and relatives in biological samples: analytical and biological challenges. *Analytical Biochemistry*.

[32] Rhee S. G., Chang T. S., Jeong W., Kang D. (2010). Methods for detection and measurement of hydrogen peroxide inside and outside of cells. *Molecules and Cells*.

[33] Li R., Huang Z., Luo J., Luo H., Wang W. (2020). Downregulation of the CB1-mediated endocannabinoid signaling underlies D-galactose-induced memory impairment. *Frontiers in Molecular Neuroscience*.

[34] Szilagyi J. T., Mishin V., Heck D. E. (2016). Selective targeting of heme protein in cytochrome P450 and nitric oxide synthase by diphenyleneiodonium. *Toxicological Sciences*.

[35] Ni S., Li D., Wei H., Miao K.-S., Zhuang C. (2021). PPARgamma attenuates interleukin-1beta-induced cell apoptosis by inhibiting NOX2/ROS/p38MAPK activation in osteoarthritis chondrocytes. *Oxidative Medicine and Cellular Longevity*.

[36] Kim T. W., Hong D. W., Park J. W., Hong S. H. (2020). CB11, a novel purine-based PPARɣ ligand, overcomes radio-resistance by regulating ATM signalling and EMT in human non-small-cell lung cancer cells. *British Journal of Cancer*.

[37] Monteghirfo S., Tosetti F., Ambrosini C. (2008). Antileukemia effects of xanthohumol in Bcr/Abl-transformed cells involve nuclear factor-kappaB and p53 modulation. *Molecular Cancer Therapeutics*.

[38] Liu N., Huang H., Liu S. (2014). Calcium channel blocker verapamil accelerates gambogic acid-induced cytotoxicity via enhancing proteasome inhibition and ROS generation. *Toxicology In Vitro*.

[39] Rastogi N., Gara R. K., Trivedi R. (2014). (6)-Gingerolinduced myeloid leukemia cell death is initiated by reactive oxygen species and activation of miR-27b expression. *Free Radical Biology & Medicine*.

[40] Skoupa N., Dolezel P., Ruzickova E., Mlejnek P. (2017). Apoptosis induced by the curcumin analogue EF-24 is neither mediated by oxidative stress-related mechanisms nor affected by expression of main drug transporters ABCB1 and ABCG2 in human leukemia cells. *International Journal of Molecular Sciences*.

[41] Uchihara Y., Tago K., Taguchi H. (2018). Taxodione induces apoptosis in BCR-ABL-positive cells through ROS generation. *Biochemical Pharmacology*.

[42] Liu X., An L. J., Li Y. (2019). Xanthohumol chalcone acts as a powerful inhibitor of carcinogenesis in drug-resistant human colon carcinoma and these effects are mediated via G2/M phase cell cycle arrest, activation of apoptotic pathways, caspase activation and targeting Ras /MEK/ERK pathway. *Journal of BUON*.

[43] Daiber A., di Lisa F., Oelze M. (2017). Crosstalk of mitochondria with NADPH oxidase via reactive oxygen and nitrogen species signalling and its role for vascular function. *British Journal of Pharmacology*.

[44] Fukai T., Ushio-Fukai M. (2020). Cross-talk between NADPH oxidase and mitochondria: role in ROS signaling and angiogenesis. *Cell*.

