# Supplementary material for: Xanthohumol Induces ROS through NADPH Oxidase, Causes Cell Cycle Arrest and Apoptosis
Source: Oxid Med Cell Longev. 2021 Nov 10;2021:9877170. doi: 10.1155/2021/9877170 (PMC8598356; doi:10.1155/2021/9877170)
Supplement: Supplementary Materials — Figure S1: the effect of DPI (10 μM) and L-NMMA (100 μM) on ROS generation in HeLa cells after 10 μM XN treated for 1 h (n = 3; ∗∗∗p < 0.001 vs. control; ###p < 0.001, “XN + DPI” vs. “XN”). Figure S2: the effect of XN on gp91phox and p47phox expression after 24 h treatment detected with Western blot and analyzed with the ImageJ software. HL-60 cells were treated with different concentrations of XN for 24 h (n = 4; ∗p < 0.05 vs. control). [file 9877170.f1.docx]

**Supplementary Materials:**

# Materials and Methods

## 1.1. Intracellular ROS generation of HeLa cell detected with DCFH-DA

Intracellular ROS levels were assessed by measuring the oxidative conversion of cell permeable DCFH-DA to fluorescent dichlorofluorescein (DCF). HeLa cells were seeded in 6-well cell culture plate. After 24 h incubation, cells were washed twice with RPMI-1640 to remove serum, and incubated with DCFH-DA (5 μM) and DPI (10 μM) or L-NMMA (100 μM) for 45 min (loaded with the florescent probe and inhibitors), then treated with XN for 1 h. Cells were then washed with PBS and fluorescent images were captured under fluorescent microscopy. The fluorescence density was quantified with the Image J software (NIH, USA).

## 1.2. Western blot analysis

NOX subunits, gp91^phox^ and p47^phox^, were detected with Western blot assay following the conventional protocol. Cells were treated with XN for 24 h, then were collected, washed with PBS, and cell lysate was prepared with RIPA Lysis Buffer. Protein concentrations were measured with the Enhanced BCA Protein Assay Kit.

# Results

XN induced ROS generation in HeLa cells, which can be inhibited with NOX inhibitor DPI, but not by NOS inhibitor L-NMMA (Fig. S1). This result confirmed our findings in HL-60 cells with a different ROS detection method.





Fig. S1. The effect of DPI (10 μM) and L-NMMA (100 μM) on ROS generation in HeLa cells after 10 μM XN treated for 1 h (n = 3; *** *p* < 0.001 vs. control; ### *p* < 0.001, “XN+DPI” vs. “XN”).

After treated by XN for 24 h, the NOX subunit gp91^phox^ was upregulated in a dose-dependent manner, while p47^phox^ was not upregulated (Fig. S2).





Fig. S2. The effect of XN on gp91^phox^ and p47^phox^ expression after 24 h treatment detected with Western blot and analyzed with Image J software. HL-60 cells were treated with different concentrations of XN for 24 h. (n = 4; * *p* < 0.05 vs. control)
